# The Xyloglucan Endotransglucosylase/Hydrolase Gene *XTH22/TCH4* Regulates Plant Growth by Disrupting the Cell Wall Homeostasis in *Arabidopsis* under Boron Deficiency

**DOI:** 10.3390/ijms23031250

**Published:** 2022-01-23

**Authors:** Cheng Zhang, Mingliang He, Zhexuan Jiang, Lan Liu, Junbao Pu, Wenjun Zhang, Sheliang Wang, Fangsen Xu

**Affiliations:** 1 National Key Laboratory of Crop Genetic Improvement, Huazhong Agricultural University, Wuhan 430070, China; zcheng93@webmail.hzau.edu.cn (C.Z.); bghy@webmail.hzau.edu.cn (M.H.); 2 Microelement Research Center/Key Laboratory of Arable Land Conservation (Middle and Lower Reaches of Yangtze River), Ministry of Agriculture, Huazhong Agricultural University, Wuhan 430070, China; jiangzhexuan@webmail.hzau.edu.cn (Z.J.); lanliu@webmail.hzau.edu.cn (L.L.); 3 College of Resources and Environment, Huazhong Agricultural University, Wuhan 430070, China; junbaopu@163.com (J.P.); wenjunzhang@mail.hzau.edu.cn (W.Z.)

**Keywords:** boron deficiency, *TCH4*, cell wall, methylesterification, ROS, *Arabidopsis* growth

## Abstract

TCH4 is a xyloglucan endotransglucosylase/hydrolase (XTH) family member. Extensive studies have shown that *XTHs* are very important in cell wall homeostasis for plant growth and development. Boron (B), as an essential micronutrient for plants, plays an essential role in the cross-linking of cell wall pectin. However, the effect of B on cell wall organization is unclear. This study aimed to explore the mechanism of plant adaption to B stress by investigating the role of *TCH4* in cell wall homeostasis. We conducted both plate and hydroponic cultures of wild-type Col-0 and overexpression and gene knockout lines of *XTH22/TCH4* to analyze the phenotype, components, and characteristics of the cell wall using immunofluorescence, atomic force microscopy (AFM), and transmission electron microscopy (TEM). B deficiency induces the expression of *TCH4*. The overexpression lines of *TCH4* presented more sensitivity to B deficiency than the wild-type Col-0, while the knockout lines of *TCH4* were more resistant to low B stress. Up-regulation of *TCH4* influenced the ratio of chelator-soluble pectin to alkali-soluble pectin and decreased the degree of methylesterification of pectin under B-deficient conditions. Moreover, we found that B deficiency disturbed the arrangement of cellulose, enlarged the gap between cellulose microfibrils, and decreased the mechanical strength of the cell wall, leading to the formation of a thickened and deformed triangular region of the cell wall. These symptoms were more profound in the *TCH4* overexpression lines. Consistently, compared with Col-0, the O_2_^−^ and MDA contents in the *TCH4* overexpression lines increased under B-deficient conditions. This study identified the B-deficiency-induced *TCH4* gene, which regulates cell wall homeostasis to influence plant growth under B-deficient conditions.

## 1. Introduction

Boron (B) is an essential micronutrient for plant growth. B deficiency causes some physiological changes, such as increased ROS and anthocyanin accumulation [1]. In addition, B deficiency also leads to many morphological defects, such as inhibition of leaf expansion, root elongation, and a reduction in fertility and crop yield [2,3,4]. B deficiency is a severe agricultural problem worldwide [5]. Many studies have indicated that the apparent function of B is its participation in the formation of the primary cell wall via cross-linking two monomeric rhamnogalacturonan II (RG-II) of the pectin to form dimeric RG-II [6,7]. The primary cell wall mainly includes cellulose, hemicellulose, and pectin. To date, there are few documents about the relationship between B and other cell wall components.

Cellulose is composed of many (1-4)-linked β-D-glucans, which form linear chain molecules through 1,4 glycosidic bonds. Many of these glucans are arranged in parallel to form cellulose microfibrils [8]. Hemicellulose is a linear polysaccharide often accompanied by short side chains that bind cellulose microfibrils by hydrogen bonds to form a resilient network [8,9,10]. In dicotyledons, such as *Arabidopsis*, xyloglucan is the main hemicellulose component of the primary cell wall [9,10], while in monocotyledons, such as maize and rice, glucuronoarabinoxylan accounts for a major proportion [11,12]. Pectin is a complex polysaccharide, mainly composed of rhamnogalacturonan I and homogalacturonan. In addition, pectin also contains smaller amounts of arabinan, xylogalacturonan, arabinogalactan I, and rhamnogalacturonan II [8]. Pectin is linked together by covalent bonds [13] and is bound to xyloglucan by covalent and non-covalent bonds [14,15]. The integration of the newly synthesized xyloglucan is attributed to the function of the xyloglucan endotransglucosylase/hydrolase (XTH) family [16]. XTH is a collection of enzymes, including xyloglucan endotransglucosylase (XET) and xyloglucan endohydrolase (XEH), which play an important role in modifications of the cell wall [17,18]. XETs are the primary cell wall enzymes that can cleave the main chain of xyloglucan and connect one end of the cleaved chain to the free end of another xyloglucan [17], and XEHs can catalyze the hydrolysis of xyloglucan [19].

Studies have shown that many *XTH* genes may be involved in cell wall modification [20,21,22,23,24]. For example, AtXTH3, a cell wall enzyme, catalyzes covalent cross-linking between cellulose and cello-oligosaccharide [24]. *XTHs* are also closely related to abiotic stress. It was reported that *XTH17* and *XTH31* regulate aluminum toxicity tolerance in *Arabidopsis* roots [18,25]. *XTH31* governs the content of cell wall xyloglucan and the capacity for binding to Al, which affects Al sensitivity [18]. XTH17 and XTH31 might co-regulate the Al-binding capacity existing as a dimer [25]. Moreover, *XTHs*, such as *XTH19*, *XTH23*, and *XTH30*, also participate in the salt-stress response [26,27]. *XTH19* and *XTH23* participate in lateral root development via the *BES1*-dependent pathway, thus affecting lateral root adaptation to salt stress [27]. *XTH30* modulates xyloglucan side chains, xyloglucan-derived oligosaccharide abundance, cellulose synthesis, and cortical microtubule stability, thus negatively regulating salt tolerance [26].

*TCH4* (*TOUCH4*), also known as *XTH22* [17], encodes a protein with an XET enzyme ability [20]. The expression level of *TCH4* is rapidly upregulated by brassinosteroids, auxins, and multiple environmental stimuli, such as touch, temperature shock, and darkness [20,28,29]. However, the knockout of *TCH4* did not show apparent growth defects. In this study, we investigated the role of *TCH4* from the perspective of cell wall modification in plants accommodating low B stress. B deficiency increased the expression of *TCH4,* which led to an altered ratio of chelator-soluble pectin to alkali-soluble pectin, decreased degree of methylesterification of pectin and mechanical strength, increased the porosity between cellulose microfibrils, disordered the arrangement of cellulose microfibrils, and, consequently, increased the sensitivity to low B stress. Collectively, we established the importance of *TCH4* in low B stress.

## 2. Results

### 2.1. TCH4 Expression Pattern and Protein Localization

**Figure 1:**
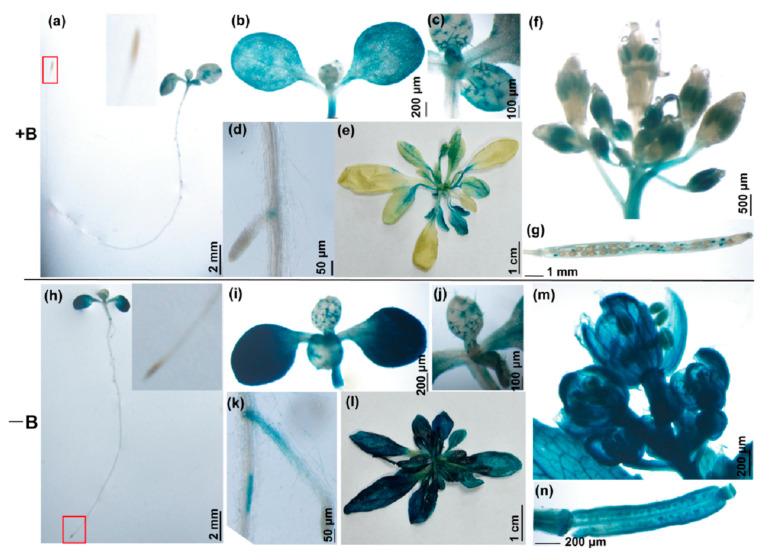
GUS staining pattern in different organs and tissues of *AtTCH4* in *Arabidopsis* under different B conditions. (**a**–**g**) and (**h**–**n**) The transgenic plants were grown for 7 d at the seedling stage and 40 d at the flowering stage under B-sufficient (100 μM B) and B-deficient (0.1 μM B) conditions, respectively. (**a**,**h**) Whole seedling plants. The root tips in the red box are enlarged. (**b**,**i**) Cotyledon. (**c**,**j**) Leaf trichome. (**d**,**k**) Lateral root node. (**e**,**l**) Rosette leaf. (**f**,**m**) Flower. (**g**,**n**) Siliques.

To investigate the expression pattern of the *TCH4* gene, a plant expressing β-glucuronidase (GUS) under the control of the *TCH4* native promoter was generated. The tissue-specific localization of *TCH4* was performed using GUS staining. Apparent GUS staining in the transgenic plants were observed in the cotyledons (Figure 1a,b), hypocotyls (Figure 1b), leaf trichomes (Figure 1c), and lateral root node (Figure 1d) at the seedling stage. Young expanding tissues of rosette leaves (Figure 1e), leaves (Supplementary Figure S1a), and siliques (Figure 1g) presented a distinguishable expression at the reproductive stage. The anther consistently showed GUS activity at the flower developmental stage 12–13 (Figure 1f). GUS activity was significantly induced in B-limited plants (Figure 1h–n; Supplementary Figure S1a–c).

**Figure 2:**
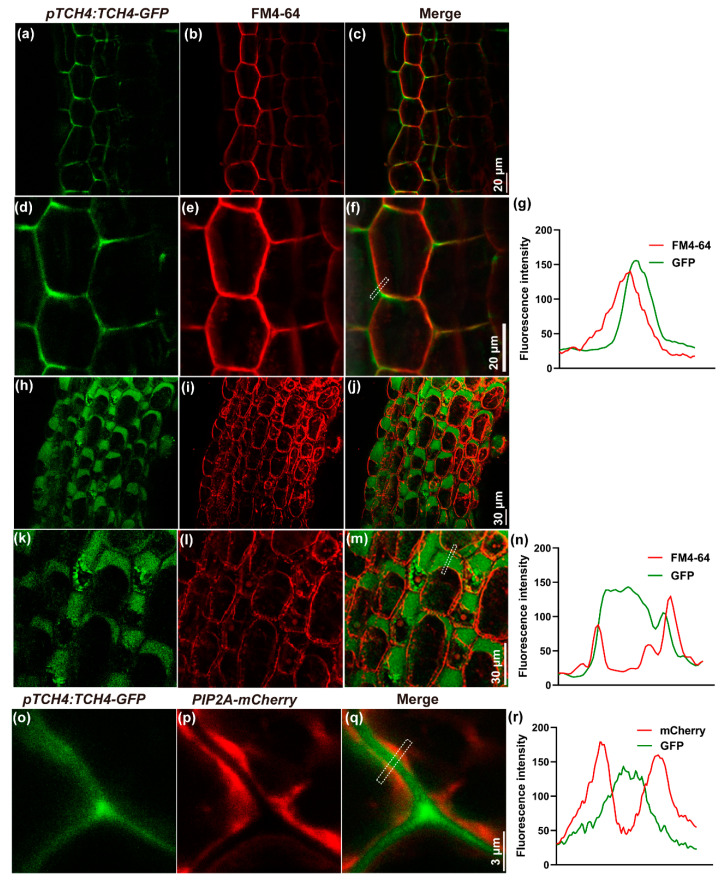
Subcellular localization of *AtTCH4* in *Arabidopsis* hypocotyls and tobacco cells. (**a**,**d**) The confocal image of *pTCH4:TCH4-GFP* fusion proteins in *Arabidopsis* hypocotyls. (**b**,**e**) FM4-64 (plasma membrane marker) localization in the same hypocotyls cells as in (**a**). (**c**,**f**) *pTCH4:TCH4-GFP* colocalized with FM4-64. (**d**–**f**) are enlarged images of (**a**–**c**), respectively. (**g**,**n**,**r**) Plot profile analysis of *pTCH4:TCH4-GFP* (green) and FM4-64 (red) signals in the marked position shown in (**f**,**m**,**q**), respectively. (**h**–**m**) The localization of (**h**,**k**) *pTCH4:TCH4-GFP*, (**i**,**l**) FM4-64, and (**j**,**m**) colocalized localization in plasmolyzed hypocotyls cells in *Arabidopsis*. (**k**–**m**) are enlarged images of (**h**–**j**), respectively. (**o**–**q**) The localization of (**o**) *pTCH4:TCH4-GFP*, (**p**) *PIP2A-mCherry*, and (**q**) colocalized localization in plasmolyzed tobacco cells.

To clarify the subcellular localization of TCH4, *pTCH4:TCH4-GFP* transgenic *Arabidopsis* was established. A strong expression of the GFP signal was observed at the junction sites delimiting the intercellular spaces and was partially overlapped by the plasma membrane (PM) dye FM4-64 (Figure 2a–g). To further clarify the cell wall or PM localization of TCH4, the plasmolysis of cells was performed, and the GFP signal was exclusively retained in the space between the PMs (Figure 2h–n). A consistent result was confirmed when it was transiently expressed in tobacco leaves (Figure 2o–r). These results indicated that *TCH4* is a B-deficiency responsive gene that encodes a cell wall-located protein.

### 2.2. TCH4 Regulates Plant’s Boron Response Independent of B Uptake

**Figure 3:**
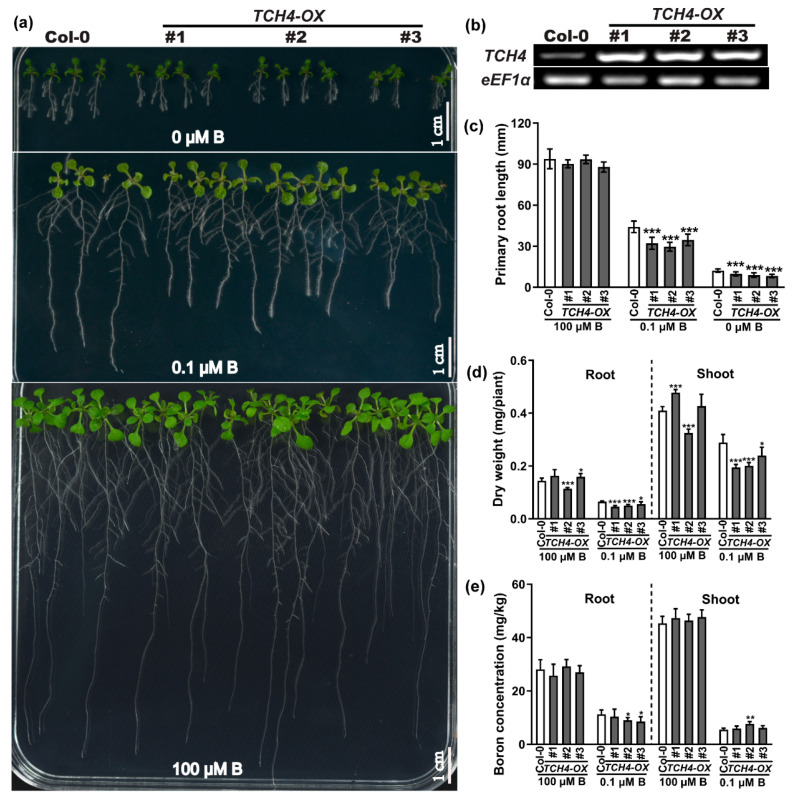
Phenotype of the *TCH4* overexpression (*TCH4*-*OX*) lines under different boron levels. (**a**) Phenotype of wild-type (Col-0) and *TCH4*-*OX* lines grown under different B concentrations. Scale bar, 10 mm. (**b**) Analysis of *TCH4* expression level by RT-PCR. (**c**) Primary root length of wild-type (Col-0) and *TCH4*-*OX* lines grown as in (**a**) (mean ± s.d., *n* = 15). (**d**) Dry weight of wild-type (Col-0) and *TCH4*-*OX* lines grown in media and supplemented with 0.1 and 100 μM B for 10 days (mean ± s.d., *n* = 7). (**e**) B concentration of Col-0 and *TCH4*-*OX* lines (mean ± s.d., *n* = 5). The asterisks indicate statistically significant differences (* *p* < 0.05, ** *p* < 0.01, *** *p* < 0.001 according to a two-tailed unpaired Student’s *t*-test).

To determine whether *TCH4* is involved in B-deficiency stress responses, three knockout lines of *TCH4* (CR#1 carried a 1 bp insertion; CR#2 carried a 34 bp deletion; and CR#3 carried a 13 bp deletion) (Supplementary Figure S2a) were established by the CRISPR/Cas9 gene-editing system [30]. The wild-type and knockout plants had no significant differences in growth phenotype, primary root length, fresh weight, and dry weight regardless of B conditions at the seedling stage (Supplementary Figure S2b–e). There are 33 members of the XTH family in *Arabidopsis thaliana* [17]. The functional redundancy between *TCH4* and other homologous genes cannot be ignored. We also established the overexpression lines of *TCH4* in a wild-type Col-0 background (Figure 3b; Supplementary Figure S3). No evident difference in growth phenotype was observed between the wild-type and *TCH4* overexpression (*TCH4*-*OX*) plants grown in normal B conditions (100 µM B) (Figure 3a). In contrast, the *TCH4*-*OX* plants presented more sensitivity to B-deficiency stress than the wild-type plants, showing reduced primary root length and dry weight (Figure 3a,c,d). Interestingly, *TCH4*-*OX* did not significantly affect the B concentration or other elemental concentrations in the plants (Figure 3e; Supplementary Figure S4). The results suggest that the distinct phenotype of *TCH4*-*OX* lines under B deficiency might be attributed to its function in cell wall modification.

### 2.3. TCH4 Participates in the Reproductive Growth Regulation of Arabidopsis under B Deficiency

**Figure 4:**
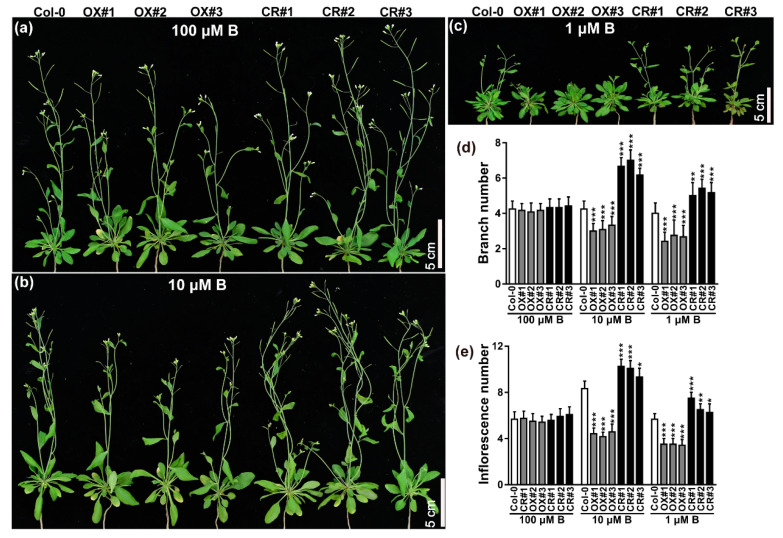
Phenotype response of the *TCH4* transgenic lines at the flowering stage to B deficiency. (**a**–**c**) Phenotype of wild-type (Col-0) and *TCH4* transgenic lines plants grown in media consisting of 100, 10, and 1 μM B for 30 d, respectively. Scale bar, 5 cm. (**d**) Number of branches and (**e**) inflorescences of Col-0 and *TCH4* transgenic lines grown as in (**a**–**c**) (mean ± s.d., *n* = 14). The branch number refers to the sum of the branches that grew out from the rosette base and the primary branches on the stem. The inflorescence number refers to all the branches that grew out from both the rosette base and branches, including primary branches, secondary branches, and tertiary branches. The asterisks indicate statistically significant differences (* *p* < 0.05, ** *p* < 0.01, *** *p* < 0.001 according to a two-tailed unpaired Student’s *t*-test).

**Figure 5:**
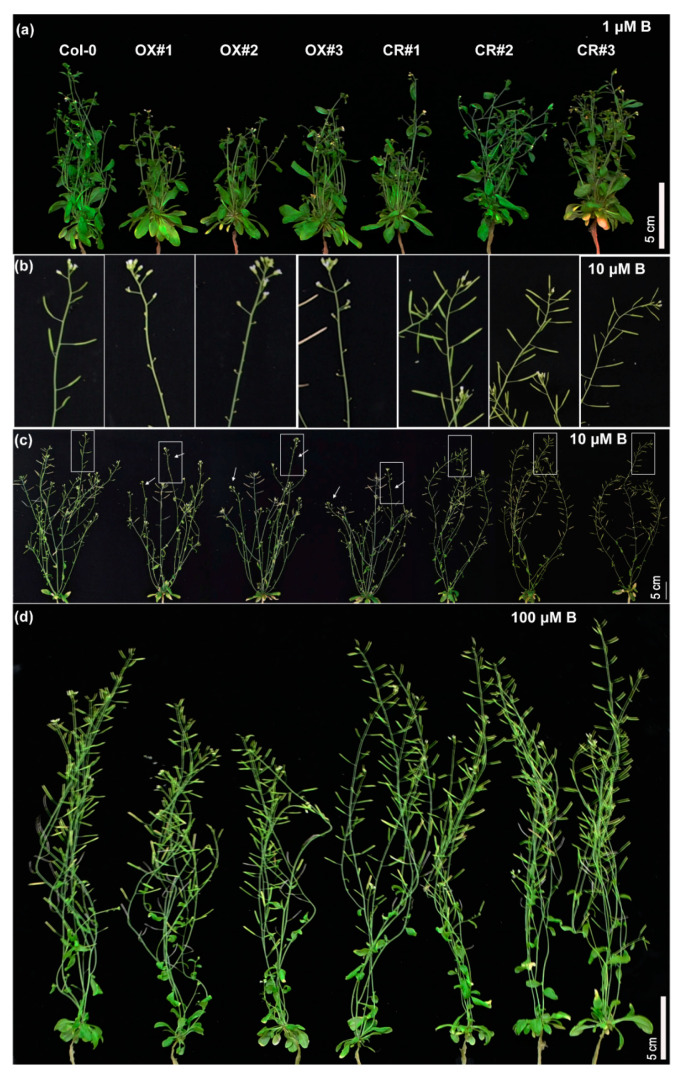
Phenotype response of the *TCH4* transgenic lines at the maturity stage. (**a**,**c**,**d**) Phenotype of wild-type (Col-0) and *TCH4* transgenic lines grown in media consisting of 1, 10, and 100 μM B for 45 d, respectively. Scale bar, 5 cm. (**b**) Close-up views of the white box in (**c**).

Considering the abundant expression of *TCH4* in flowers and siliques, we assessed the effect of *TCH4* activity on plant responses to B deficiency at the reproductive stage. The Col-0 and *TCH4* transgenic lines were grown hydroponically under various B conditions during the reproductive stage for 30 d and 45 d, respectively. The shoot growth of all plants was inhibited under B-limited conditions relative to adequate B conditions (Figure 4a–c and Figure 5a–d). The *TCH4-OX* lines presented fewer branches and inflorescences than Col-0, while the *TCH4* knockout lines developed more branches and inflorescences than Col-0 under 10 and 1 µM B conditions (Figure 4a–e). All plants developed a comparable number of branches and inflorescences at 100 µM B (Figure 4a,d,e). In particular, 1 µM B was not sufficient to complete the entire life cycle for all plants, leading to infertility (Figure 5a). At maturity, all plants can grow normally under 100 µM B, but not under 1 µM B (Figure 5a,d). At 10 µM B, the *TCH4-OX* lines hardly had normal siliques, while Col-0 grew normal siliques (Figure 5b,c). Under this condition, the *TCH4* knockout lines presented a large number of siliques (Figure 5b,c). These results indicate that *TCH4* participates in the reproductive growth regulation of *Arabidopsis* under B deficiency.

### 2.4. TCH4 Functions in Pectin Modification under B Deficiency

**Figure 6:**
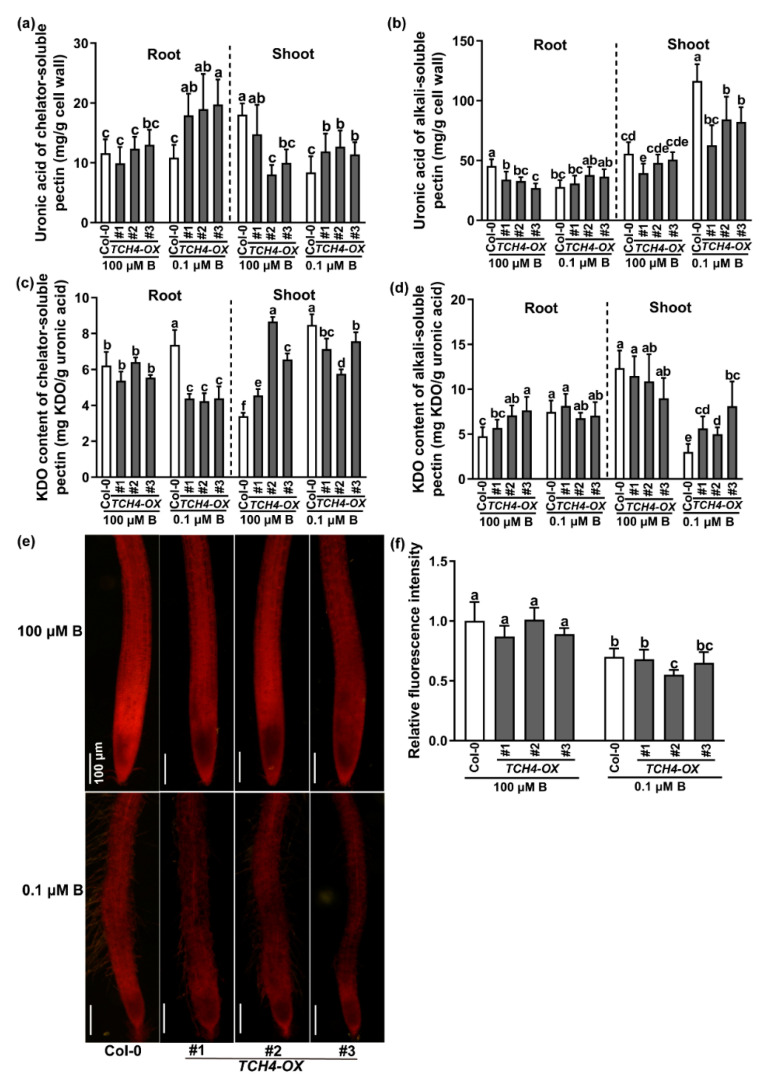
Differences in pectin content between Col-0 and *TCH4*-*OX* plants. Plants were grown in solid media with normal B (100 μM) for 8 d and then transferred to a nutrient solution containing 100 and 0.1 μM B until bolting. (**a**,**b**) The uronic acid and (**c**,**d**) KDO content were measured in (**a**,**c**) chelator-soluble pectin and (**b**,**d**) alkali-soluble pectin. (**e**) Col-0 and *TCH4*-*OX* plants grown in media consisting of 100 and 0.1 μM B conditions for 10 d. Ruthenium red (RR) staining indicates the content of pectin. Scale bar, 100 μm. (**f**) Relative fluorescence intensity was measured in the seedlings grown as in (**e**) (means ± s.d., *n* = 4). The different letters above the columns indicate significant differences between all genotypes and all growth conditions (*p* ≤ 0.05).

We analyzed the alteration of the cell wall component between Col-0 and TCH4-OX plants. As shown in Supplementary Figure S5a, the B-deficiency treatment increased the cell wall extraction ratio in both roots and shoots compared to the adequate B treatment, and the cell wall extraction rate was higher in the TCH4-OX plants than in wild-type plants under B-deficient conditions, especially in the roots. The contents of cellulose and hemicellulose showed no significant differences between Col-0 and TCH4-OX plants (Supplementary Figure S5b,c). Interestingly, the content of chelator-soluble pectin in the roots and shoots of the TCH4-OX plants was higher than that in the wild-type plants under B-deficient conditions (Figure 6a,b). In comparison, the content of alkali-soluble pectin in the shoots was lower than that in the wild-type plants (Figure 6a,b). Moreover, ruthenium red (RR) staining showed no significant difference in total pectin content in the roots between the wild-type Col-0 and TCH4-OX plants regardless of B conditions (Figure 6e,f). The results indicate that TCH4 specifically affected the ratio of chelator-soluble pectin to alkali-soluble pectin under B-deficient conditions.

2-keto-3-deoxyoctonic acid (KDO) can be used to indicate the content of RG-II in cell wall pectin [31]. We further measured the content of KDO in chelator-soluble and alkali-soluble pectin. The data revealed that the KDO content of chelator-soluble pectin was increased by the B-deficient treatment in the wild-type Col-0 but not in TCH4-OX plants (Figure 6c). B-deficient treatment increased the KDO content of alkali-soluble pectin in the roots of wild-type Col-0 plants, while it decreased the KDO content of alkali-soluble pectin in the shoots of wild-type Col-0 plants (Figure 6d). The B concentrations had no significant effect on the KDO content of alkali-soluble pectin in the roots of TCH4-OX lines (Figure 6d). In contrast, B deficiency alleviated the reduction in KDO content of alkali-soluble pectin in TCH4-OX shoots compared to wild-type Col-0 plants (Figure 6d).

**Figure 7:**
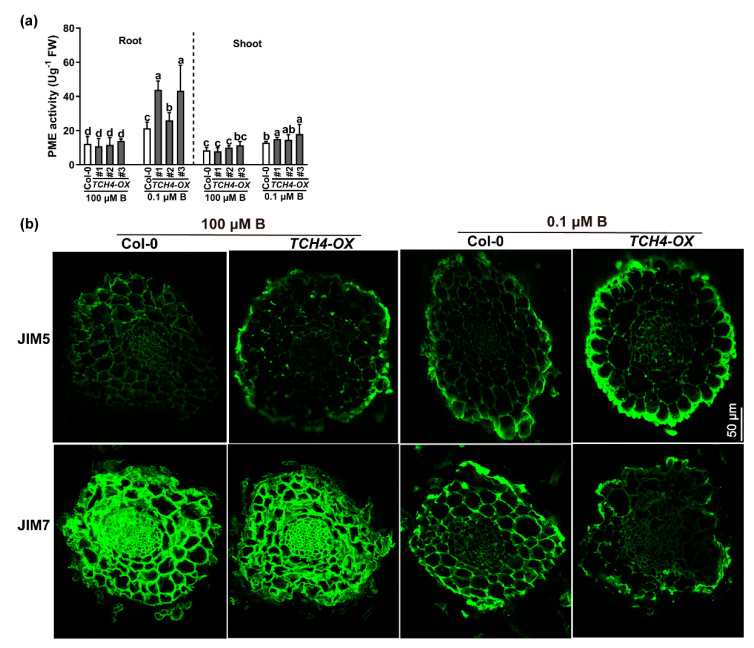
Differences in the degree of pectin methylesterification between Col-0 and *TCH4*-*OX* plants. (**a**) Differences in PME enzyme activities between Col-0 and *TCH4*-*OX* plants (means ± s.d., *n* = 5). (**b**) Immunohistochemical analysis in root cross-sections of Col-0 and *TCH4*-*OX* plants. Scale bar, 50 μm. The different letters above the columns indicate significant differences between all genotypes and all growth conditions (*p* ≤ 0.05).

Pectin in the nascent cell wall is highly methylated and can be demethylated by pectin methylesterase (PME) [32]. The methylesterification degree of pectin can affect the cross-linking of pectin, thus influencing the compactness of the cell wall. We therefore determined the PME activities between Col-0 and TCH4-OX plants grown under adequate and low B conditions. We found that B deficiency increased the PME enzyme activities in both the roots and shoots, and PME enzyme activities in the TCH4-OX plants were higher than those in the wild-type Col-0 plants (Figure 7a). These results indicate that B deficiency might reduce the degree of pectin methylesterification. In parallel, we performed an immunohistochemistry experiment to compare the methylesterification degree of pectin between Col-0 and TCH4-OX plants grown in solid media under adequate B and B-deficient conditions for 10 d. A cross-section of roots was tested. The JIM5 antibody and the JIM7 antibody are extensively applied to evaluate low methyl-ester pectin and high methyl-ester pectin in plants, respectively [33]. Consistent with the results of PME enzyme activity, neither JIM5 nor JIM7 significantly labeled cell wall differences between Col-0 and TCH4-OX plants under adequate B conditions, but the immune intensity of JIM7 was more potent than that of JIM5 (Figure 7b; Supplementary Figure S5d). The B-deficient treatment reduced the JIM7 immune intensity, which was reduced more in TCH4-OX plants (Figure 7b; Supplementary Figure S5d). In contrast, JIM5 immune intensity was more evident in the TCH4-OX plants than in the Col-0 plants. These results illustrate that B deficiency reduces the methylesterification degree of pectin in the roots and that TCH4 functions in pectin demethylesterification.

### 2.5. TCH4 Influences the Cell Wall’s Spatial Structure

**Figure 8:**
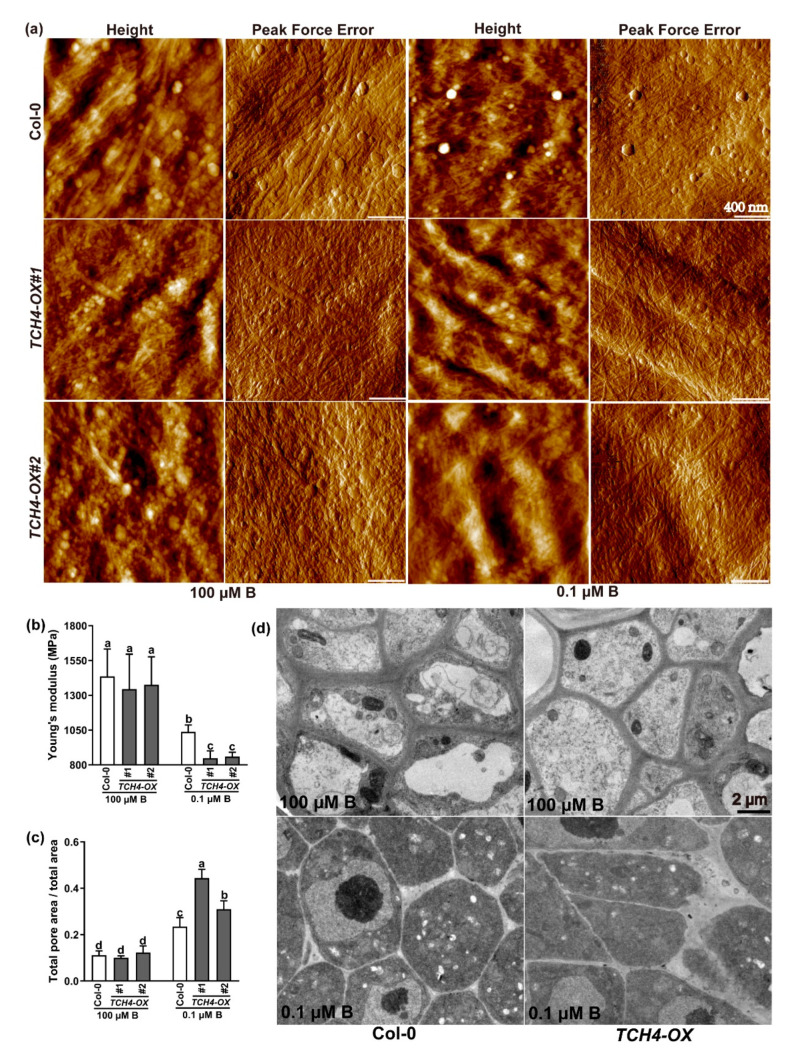
Cell wall characteristics of Col-0 and *TCH4*-*OX* lines in roots. The plants were grown in media consisting of 100 μM B and 0.1 μM B for 10 d. (**a**) Atomic force microscopy (AFM) revealing the cell wall’s surface characteristics and topography of the Col-0 and *TCH4*-*OX* lines in roots. The height and peak force error images are presented. Scale bar, 400 nm. (**b**,**c**) Young’s modulus (**b**) and the ratio of total pore area versus the total area were measured (**c**). The different letters above the columns indicate significant differences between all genotypes and all growth conditions (*p* ≤ 0.05). (**d**) Transmission electron microscopy (TEM) analysis of root meristem cells. Scale bar, 2 μm.

Pectin alteration by TCH4 function might subsequently influence the cross-linking of cell wall components and eventually cause the cell wall’s spatial structure to change. To this end, atomic force microscopy (AFM) was employed to explore the cell wall surface characteristics of Col-0 and TCH4-OX lines in roots. Under adequate B conditions, the height images showed that Col-0 and TCH4-OX lines had a natural arrangement of cellulose microfibrils with some small pores (dark colors) (Figure 8a). The ratios of the total pore area versus total area between Col-0 and TCH4-OX lines were not significant under adequate B conditions (Figure 8c). However, the ratios of the total pore area versus the total area were significantly greater in the B-deficient conditions compared with the adequate B conditions and were higher in the TCH4-OX lines than in Col-0 (Figure 8a,c).

Moreover, the mechanical strength of the cell wall was characterized by Young’s modulus. Under adequate B conditions, no apparent difference in Young’s modulus was found in the roots of any of the plants (Figure 8b). However, B deficiency attenuated Young’s modulus, which was lower in the TCH4-OX lines than in Col-0 (Figure 8b). Transmission electron microscopy (TEM) of root meristem cells showed that the junction sites delimiting the intercellular spaces were thickened and deformed under B-deficient conditions compared with normal B conditions (Figure 8d). The TCH4-OX lines developed thicker and more deformed junction sites, delimiting the intercellular spaces, than Col-0 under deficient-B conditions (Figure 8d). The above results indicate that B deficiency disturbs the cell wall’s spatial structure, and overexpression of TCH4 likely accentuates these defects.

### 2.6. TCH4 Functions in ROS Accumulation under B Deficiency

**Figure 9:**
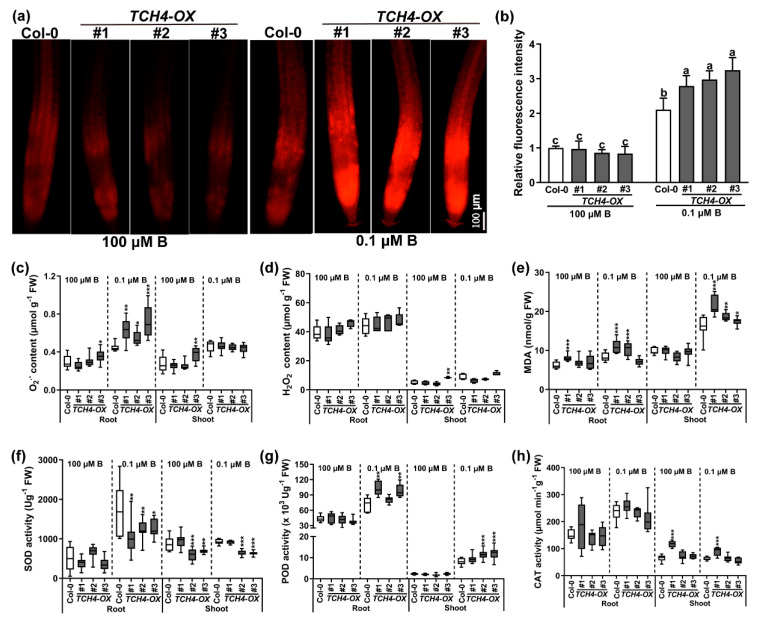
Differences in O_2_^−^, H_2_O_2_, and MDA content and antioxidant enzyme activities in Col-0 and TCH4-OX plants. The plants were grown in media consisting of 100 and 0.1 μM B conditions for 10 d. (**a**) DHE staining. Scale bar, 100 μm. (**b**) Relative fluorescence intensity was measured (means ± s.d., *n* = 7). The different letters above the columns indicate significant differences between all genotypes and all growth conditions (*p* ≤ 0.05). (**c**) O_2_^−^, (**d**) H_2_O_2_, (**e**) MDA content, (**f**) SOD, (**g**) POD, and (**h**) CAT activity were determined (mean ± s.d., *n* = 6). The asterisks indicate statistically significant differences (* *p* < 0.05, ** *p* < 0.01, *** *p* < 0.001 according to a two-tailed unpaired Student’s *t*-test).

The accumulation of ROS is often accompanied by stress in plants. Hence, we analyzed the content of free oxygen radicals (O_2_^−^) and hydrogen peroxide (H_2_O_2_) in the Col-0 and *TCH4*-*OX* plants. Compared with adequate B conditions, the content of O_2_^−^ increased in the roots, and the overexpression of *TCH4* abundance intensified this under B-deficiency stress (Figure 9a,b). The content of O_2_^−^ was further quantitatively determined. A similar result to that of DHE staining was observed in the roots (Figure 9c), while comparable O_2_^−^ contents existed in the shoots of the Col-0 and *TCH4*-*OX* plants regardless of B conditions (Figure 9c). The *TCH4* function hardly regulated the homeostasis of H_2_O_2_, although some *TCH4*-*OX* plants showed a higher H_2_O_2_ content than that of Col-0 (Figure 9d). Malondialdehyde (MDA) is considered to be the product of lipid peroxidation; thus, it is an indicator of membrane lipid oxidation, reflecting tissue damage by B-deficiency stress to a certain extent [34]. Under adequate B conditions, no apparent difference in the MDA content of the plants was detected (Figure 9e). In contrast, B deficiency elevated the MDA content, and the MDA content of the *TCH4*-*OX* lines was higher than that of Col-0 under low B stress (Figure 9e).

An antioxidant enzyme system can eliminate ROS to avoid excessive accumulation of ROS in plants. Therefore, the superoxide dismutase (SOD), peroxidase (POD), and catalase (CAT) enzyme activities were measured to explore the stress experienced by the plants. No apparent differences were detected in the tested plants under normal-B conditions (Figure 9f–h). However, B deprivation notably advanced the SOD, POD, and CAT enzyme activities. The *TCH4*-*OX* lines displayed decreased SOD activity and increased POD activity compared with Col-0 under low B stress (Figure 9f,g). No significant difference was found in CAT activity in the Col-0 and *TCH4*-*OX* lines (Figure 9h). The results showed that *TCH4*-*OX* lines accumulated more O_2_^−^ and had a weaker ability to scavenge O_2_^−^ than Col-0 under B-deficient conditions.

## 3. Discussion

Previous studies have shown that xyloglucan is a substrate of TCH4; thus, TCH4 probably plays a vital role in cell wall modification [20,35]. Here, by viewing the intracellular localization of the TCH4-GFP protein, we validated that *TCH4* is a cell wall-localized enzyme (Figure 2). The plant cell wall is a complex and dynamic structure and provides structural support for plant growth and development. A large number of studies have shown that cellulose is the main framework of the cell wall and have revealed the significance of hemicellulose and pectin in plant growth and development [36]. The rapid remodeling of cell wall components by multiple genes confers flexibility in coping with developmental and various stressors. Nuclear magnetic resonance studies have indicated that pectins and xyloglucans account for the majority of the contact with cellulose surfaces [37], suggesting that modifying one component in the cell wall may affect the others. For example, the mutation of *QUA2,* a pectin methyltransferase, affects cellulose biosynthesis [38], supporting the interaction of pectin and cellulose. In this study, the expression of *TCH4* did not significantly change the contents of cellulose and hemicellulose (Supplementary Figure S5b,c). However, the ratio of chelator-soluble pectin and alkali-soluble pectin, as well as the KDO-labeled RG-II level, were disrupted by *TCH4* overexpression (Figure 6a–d). Meanwhile, the distinct methylesterification degree and cellulose arrangement in Col-0 and TCH4-OX plants were evident (Figure 7b and Figure 8a). These differences might facilitate homeostasis disorders of the cell wall in TCH4-OX plants under B-deficient conditions, thus showing worse development than Col-0. 

Several studies have reported that the genetic deletion of xyloglucan synthesis-related enzymes did not significantly disable cell wall functions or the phenotype. For example, the loss of function of two xylosyltransferase genes, *XXT1* and *XXT2*, caused a slight decrease in the xyloglucan content without morphological phenotype alteration [39]. *AtXLT2,* which participates in xyloglucan biosynthesis, belongs to the same subclade of the glycosyltransferase family 47 and *MUR3*. The double mutant of *mur3 xlt2* only showed relatively lighter defects with slight dwarfism [40]. Glucuronyltransferase GUX participated in the addition of glucuronic acid side chains onto xylan. The phenotype of the *gux1 gux2* mutant presented no apparent differences compared with the wild type [41]. UXT1 is a UDP-xylose transporter that belongs to the NST family in *Arabidopsis thaliana*. Although the *uxt1* mutant showed a distinct decrease in Xyl and GlcA, there was no noticeable difference in morphological phenotype between the *uxt1* mutant and the wild type [42]. These can be explained either by the functional redundancy among homologous genes, the requirements of the given stress, or the given developmental stage. For example, constitutive expression of hot pepper gene CaXTH3 in tomato plants did not cause phenotype alteration, while the enhanced growth of transgenic tomato plants was observed under salt stress [43]. The Arabidopsis *XTH* family has 33 members; thus, the homologue of *TCH4* may be responsive to B regulation and may functionally complement *TCH4*, leading to invisible phenotypical differences between *tch4* mutants and Col-0 plants (Figure 3a,c; Supplementary Figure S2b,c). *TCH4* is mainly expressed in young tissues where B-deficiency symptoms preferentially occur (Figure 1; Supplementary Figure S1). Consistently, the *TCH4* knockout lines presented more branches and inflorescences under inadequate B conditions at the reproductive stage than Col-0 and *TCH4-OX* lines (Figure 4a–e). Moreover, the *TCH4* knockout lines had large and numerous siliques compared with wild-type Col-0 and *TCH4-OX* lines under inadequate B conditions (10 µM B) (Figure 5). Plants in the reproductive stage have a higher B demand than those in the seedling stage; thus, a decrease in the number of branches by *TCH4* expression is likely an effective strategy that facilitates the formation of siliques when B is inadequate. These findings indicate the requirement of *TCH4* function for plant growth in response to B deficiency at the reproductive stage.

## 4. Materials and Methods

### 4.1. Plant Materials and Growth Conditions

All wild-type and transgenic *Arabidopsis thaliana* plants in this study were from the Col-0 background. The experiments were carried out in plate cultures at the seedling stage, except that the extraction of the cell wall components and the phenotypic investigation of the flowering and maturity stages were cultured in a hydroponic system. The seeds were surface-sterilized and germinated for 10 d in media [44] consisting of 1% sucrose and 1% gellan gum supplemented with different B concentrations after two days of vernalization at 4 °C. For the phenotypic investigation of the flowering and maturity stages, the seeds were sown on solid media consisting of 100 µMB for 8–10 d and then transferred to the hydroponic culture system with Hoagland and Arnon solution [45] containing different B treatments. All plants were grown under controlled environmental conditions at 22/20 °C (light/dark), 300–320 μmol·m^−2^·s^−1^, and a 16/8 h (light/dark) photoperiod.

### 4.2. Generation of pTCH4:GUS Transgenic Lines and Histochemical Assay

In order to construct the *pTCH4:GUS* plasmid, the *AtTCH4* promoter region (about 1.7 kb) was amplified by PCR with the TCH4-GUS primer pair (Supplementary Table S1) and then inserted into the *HindIII* and *XbaI* restriction sites of a PBI121 vector. The *pTCH4:GUS* transgenic plants were obtained via the *Agrobacterium*-mediated floral-dip method [46]. GUS histochemical staining was performed as described by Gonzalez-Garcia et al. [47].

### 4.3. Subcellular Localization of AtTCH4

In order to construct the *pTCH4:TCH4-GFP* plasmid, the promoter and genome of *TCH4* were amplified via PCR with the TCH4-GFP-1 and TCH4-GFP-2 primers successively (Supplementary Table S1) and then inserted into the rebuilt PBI121 vector at *ScaI* and *SmaI* restriction sites. *pTCH4:TCH4-GFP* transgenic plants were generated via the *Agrobacterium*-mediated floral-dip method [46]. For GFP observation, plants soaked in 4 μM FM4-64 with or without 0.4 g/mL sucrose for plasmolysis were observed by a Leica TCS SP8 confocal laser scanning microscope (www.leica-microsystems.com/home/ accessed on 1 November 2021). For GFP observation in *Nicotiana benthamiana* leaves, the *pTCH4:TCH4-GFP* and *PIP2A-mCherry* plasmids [48] co-injected into tobacco leaves soaked with or without 0.4 g/mL sucrose for plasmolysis were inspected by confocal laser scanning microscopy.

### 4.4. Construction and Transformation of Overexpression (OX) and Knockout Vector

To establish *TCH4*-*OX* plants, the coding sequence of *TCH4* without a terminator was amplified by PCR with specific primers (Supplementary Table S1) and then cloned into the *AscI* and *BamHI* restriction sites of pBinGlyRed3, which contains the 35S promoter. To generate the *TCH4*-targeted mutants, a CRISPR/Cas9-based pRSE-WH vector [30] was used to carry out the target sequence CCTTTCACTGCTTCTTACCG (http://crispr.hzau.edu.cn/CRISPR/ accessed on 20 June 2019). The *Agrobacterium*-mediated floral-dip method [46] was employed in the Col-0 background.

### 4.5. RNA Extraction and Gene Expression Analysis

Plants (9 days old) of the Col-0 and *TCH4*-*OX* lines grown in media containing 100 μM B were transferred to 0.1 or 100 μM B media for 24 h. Total RNA was extracted from shoot and root samples using TRIzol (Invitrogen). Reverse transcription was implemented using ReverTra Ace qPCR RT Master Mix with a gDNA Remover kit (TOYOBO). Quantitative real-time PCR was carried out using SYBR Green PCR (TOYOBO, Osaka, Japan) with the Real-time PCR Detection System of QuantStudio^TM^6 Flex (ABI, Foster City, CA, USA). Four biological replicates and three technical replicates were performed for each sample. The relative expression levels were normalized by *UBQ5* and *eEF1α* and calculated using 2^−ΔΔCt^. The primers for qRT-PCR are shown in Supplementary Table S1.

### 4.6. Measurement of B and Other Nutrients’ Concentrations

For the measurement of B and other nutrients’ concentrations, the dried samples were ground into a powder with a carnelian mortar and were extracted with 1 M HCl by shaking (250 rpm) for 2 h. The filtered solution was determined via inductively coupled plasma-optical emission spectrophotometry (Thermo Scientific, Waltham, MA, USA).

### 4.7. Extraction and Analysis of Cell Wall Components

The cell wall was extracted according to the method of Hu and Brown [49]. Briefly, the samples were homogenized in liquid nitrogen and then washed sequentially with ultrapure water (twice), 80% ethanol (three times), a methanol chloroform mixture (1:1, *v*/*v*) (once), and acetone (once). The remaining insoluble residue was freeze-dried and defined as the cell wall, which was stored to analyze the cell wall components. Chelator-soluble pectin, alkali-soluble pectin, hemicellulose, and cellulose were extracted sequentially from the cell wall by the method described in [50]. The KDO and uronic acid content were measured colorimetrically by thiobarbituric acid [51] and hydroxydiphenyl [52], respectively. The content of hemicellulose and cellulose was analyzed via the phenol–sulfuric acid method [53].

### 4.8. Pectin and O_2_^−^ Staining

Pectin was assessed by ruthenium red (RR) [54]. The roots were immersed in 0.05% (*w*/*v*) ruthenium red dye for 15 min, washed with deionized water, and then photographed by a bright-field microscope. O_2_^−^ was determined by dihydroethidium (DHE) [55]. The roots were soaked in 10 µM DHE solution (dissolved in 10 mM Tris-HCl at pH 7.4) in the dark for 30 min and were observed using a fluorescence microscope. The relative fluorescence intensity was analyzed with ImageJ.

### 4.9. Immunofluorescence Localization of JIM5 and JIM7

The roots were fixed in 4% (*w*/*v*) paraformaldehyde for 1 h and washed in phosphate-buffered saline (PBS, pH 7.2). After incubation in a blocking solution of 0.2% (*w*/*v*) bovine serum albumin (dissolved in PBS, pH 7.2) for 1 h, the roots were incubated with the primary antibodies JIM5/JIM7 (dilution 1:10 in PBS containing 0.2% (*w*/*v*) bovine serum albumin, pH 7.2) for 3 h. After rinsing in PBS buffer, the roots were incubated in the secondary antibody (goat anti-rat IgG, dilution 1:50 in 0.2% (*w*/*v*) bovine serum albumin) for 2 h at 37 °C. The roots were washed with PBS and observed using a confocal laser scanning microscope.

### 4.10. Measurements of Antioxidant Enzyme Activities, PME Enzyme Activity, O_2_^−^, H_2_O_2_, and MDA Content

Fresh samples were homogenized in liquid nitrogen and mixed in a Tris-HCl buffer for antioxidant enzyme activity measurement (pH 7.8, 100 mM). After centrifugation at 10,000× *g* for 10 min, the supernatant was used to determine the enzyme activities of superoxide dismutase (SOD), catalase (CAT), and peroxidase (POD) [33]. For PME enzyme activity determination, the crude enzyme solution was extracted as described by Jolie et al. [56], and the PME enzyme activity was measured according to the method of Li et al. [57]. The contents of O_2_^−^ and H_2_O_2_ were measured by hydroxylamine oxidation [58] and the potassium iodide method [59], respectively. The MDA content was determined according to the method of Feng et al. [34].

### 4.11. AFM and TEM

For atomic force microscopy (AFM) observation, the cell wall was extracted from 10-day-old whole roots, and three biological repeats were tested as described by He et al. [60]. AFM was implemented as described by Zhou et al. [61]. In brief, the cell wall samples were suspended with ultra-high-purity water, and then dropped onto a clean glass slide through a pipette and dried naturally in air overnight. Different probes were used to obtain the morphology and force curves, respectively. The AFM (Bruker, Santa Barbara, CA, USA) images were obtained in the ScanAsyst-Air mode. We used Bruker ScanAsyst-Air probes with a tip radius of 2–12 nm. The cantilever was made of silicon nitride with a spring constant of 0.4 N/m. The images were obtained at a low scanning speed (1 Hz). After the imaging was completed, a harder probe was used to measure the mechanical properties (RTESP; Bruker). The tip radius of the probe was 8 nm, and the spring constant was between 20 and 80 N/m. Young’s modulus was calculated using NanoScope analysis software (Bruker) by analyzing the force curves. The total pore area/total area was analyzed by calculating the ratio of pore area to total area of each morphology via ImageJ. Transmission electron microscopy (TEM) was carried out according to Zhang et al. [62].

### 4.12. Statistical Analysis

All statistical analyses were carried out using SPSS (Statistical Package for the Social Sciences). The data were presented as means ± SD and were compared based on two-tailed unpaired Student’s t-test (* *p* < 0.05, ** *p* < 0.01, *** *p* < 0.001). For multiple comparisons, the two-way ANOVA was performed at *p* ≤ 0.05.

## 5. Conclusions

This study identified a low B responsive gene, *XTH22/TCH4*, which plays an important role in regulating the temporal and spatial growth of Arabidopsis under different B dynamics. Further investigation demonstrated that the expression of *TCH4* altered the ratio of chelator-soluble pectin to alkali-soluble pectin, the degree of methylesterification of pectin, the arrangement of cellulose, the network of cellulose microfibrils, and the mechanical strength of cell walls under B-deficient conditions. Collectively, these findings illustrate that *TCH4* regulates cell wall homeostasis under B deficiency.

## Data Availability

The data are available on request.

## Supplementary Materials

The following supporting information can be downloaded at: https://www.mdpi.com/article/10.3390/ijms23031250/s1.
