# Supplementary material for: The Xyloglucan Endotransglucosylase/Hydrolase Gene XTH22/TCH4 Regulates Plant Growth by Disrupting the Cell Wall Homeostasis in Arabidopsis under Boron Deficiency
Source: Int J Mol Sci. 2022 Jan 23;23(3):1250. doi: 10.3390/ijms23031250 (PMC8836128; doi:10.3390/ijms23031250)
Supplement: Supplementary file 1 [file ijms-23-01250-s001.zip › ijms-1500136-supplementary.pdf]

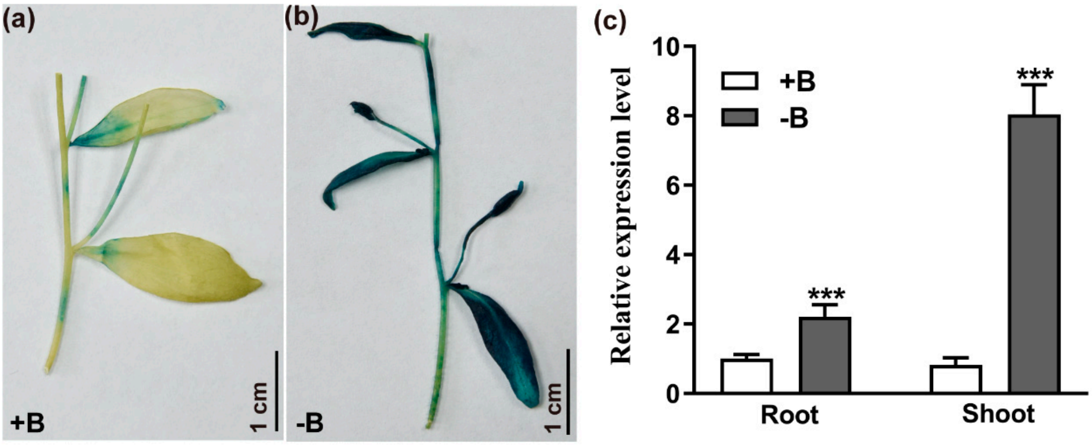

Figure S1. GUS staining pattern of *AtTCH4* at the flowering stage under different B conditions and relative expression level of *AtTCH4* in *Arabidopsis*. (a-b) The leaves of transgenic plants harboring *pTCH4: GUS* were stained for GUS activity. The plants were grown for 40 d at the flowering stage under (a) B-sufficient (100 μM B) and (b) B-deficient (0.1 μM B) conditions respectively. (c) Relative expression of *AtTCH4* in roots and shoots under B-sufficient (100 μM B) and B-deficient (0.1 μM B) conditions (mean ± s.d., n=4). The asterisks indicate statistically significant differences (***, $P < 0.001$ according to two-tailed unpaired Student's *t*-test).

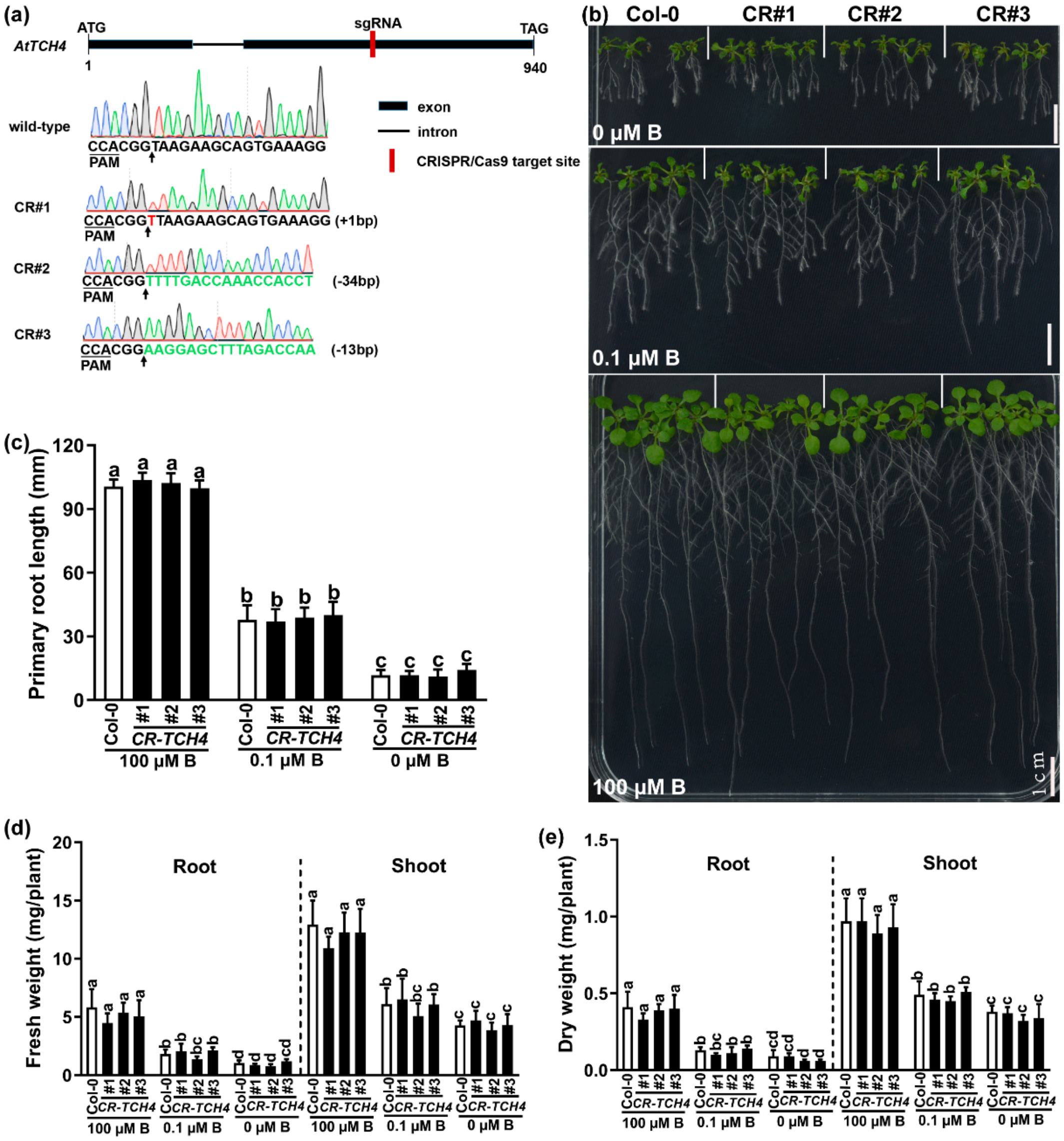

Figure S2. Phenotype of *TCH4* knockout lines. (a) CRISPR/Cas9- mediated target mutagenesis of *AtTCH4*. The edit types of three *AtTCH4* homozygous mutants (CR#1, 2, 3) are shown. (b) Phenotype of wild-type (Col-0) and *TCH4* knockout lines plants grown on media consisting of 0, 0.1 and 100 μM B for 10 d. Scale bar, 10 mm. (c) Primary root length of wild-type (Col-0) and *TCH4* knockout lines plants grown as in (b) (mean ± s.d., n = 30). (d) Fresh weight and (e) dry weight of wild-type (Col-0) and *TCH4* knockout lines plants grown as in (b) (mean ± s.d., n = 10). The different letters above the columns indicate significant differences between all the genotypes and all the growth conditions ($P \leq 0.05$).

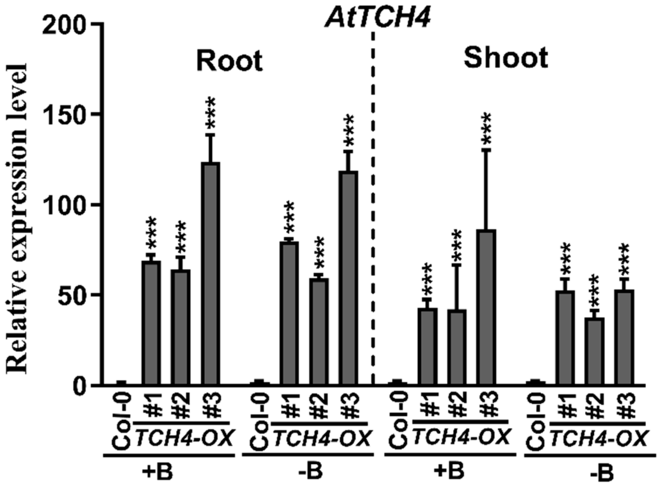

Figure S3. Relative expression of *TCH4* in roots and shoots under 100 and 0.1 µM B (mean ± s.d., n=4). The asterisks indicate statistically significant differences (***, $P < 0.001$ according to two-tailed unpaired Student's t-test).

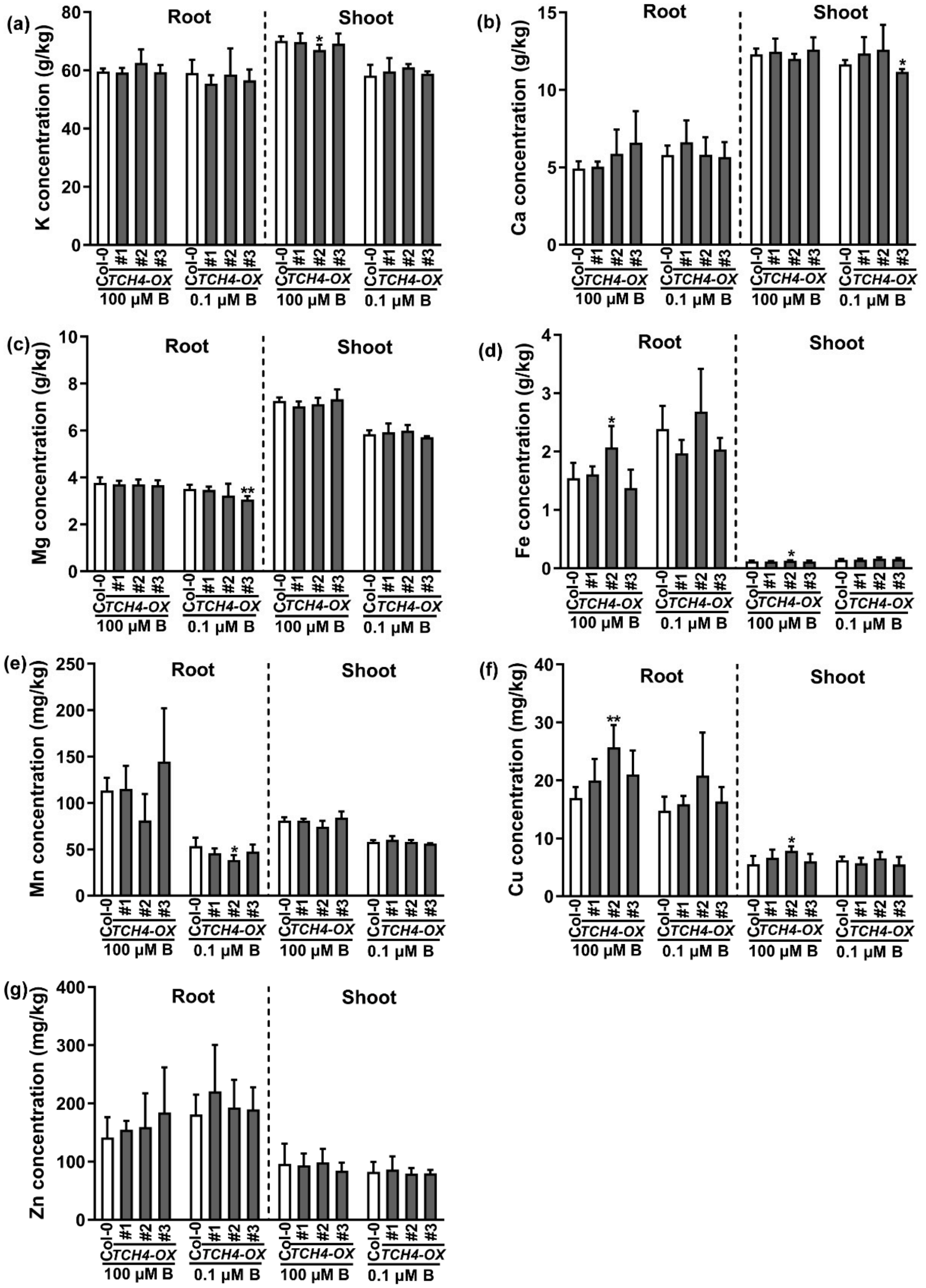

Figure S4. Elemental analysis of root and shoot between Col-0 and *TCH4-OX* lines. The plants were grown on media consisting of 100 and 0.1 μM B for 10 days. (a-g) The concentration of K (a), Ca (b), Mg (c), Fe (d), Mn (e), Cu (f), Zn (g) between Col-0 and *TCH4-OX* lines (mean ± s.d., n = 5). The asterisks indicate statistically significant differences (* $P < 0.05$, **, $P < 0.01$ according to two-tailed unpaired Student's *t*-test).

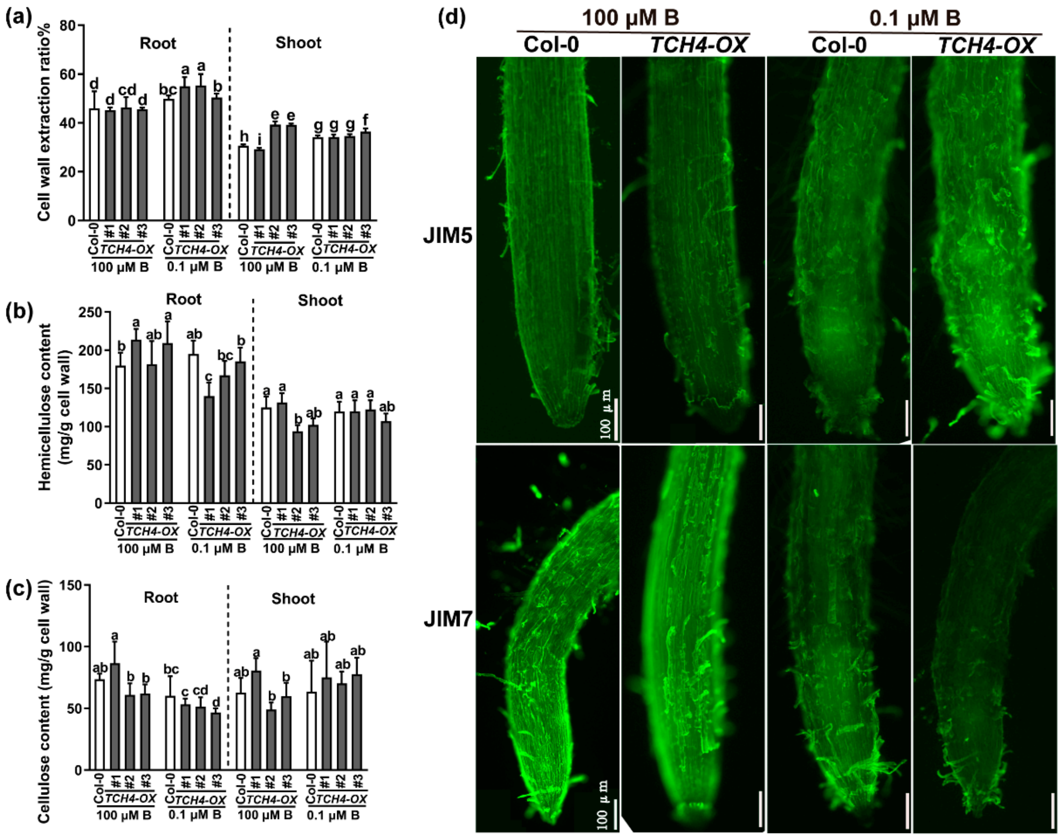

Figure S5. Differences of cell wall components and degree of methylesterification of pectins between Col-0 and *TCH4-OX* plants grown under deficient B and adequate B conditions. (a) The cell wall extraction ratio was the dry weight of cell wall divided by the total dry weight. (b) The hemicellulose content, (c) cellulose content was determined. Data are presented as mean ± s.d. There were four biological replicates in root samples and six biological replicates in shoot samples. The different letters above the columns indicate significant differences between all the genotypes and all the growth conditions ($P \leq 0.05$). (d) Immunolocalization of low methyl-ester pectin (JIM5 epitope) and high methyl-ester pectin (JIM7 epitope) in roots between Col-0 and *TCH4-OX* plant. Scale bar, 100 μm.

Table S1. *Primers used for cloning and qRT-PCR.*

Use	Primer name	Primers (5'-3')
Vector construction	TCH4-GUS-F	TGATTACGCCAAGCTTTCACGCCTGACCTAGACTTAC
	TCH4-GUS-R	CCGGGGATCCTCTAGTTTCTAGAGATTGTAGATATTCT TGTATTGAG
	TCH4-GFP-F1	TCACGCCTGACCTAGACTTACTGG
	TCH4-GFP-R1	TGCAGCTAAGCACTCTTTAGGAAGACC
	TCH4-GFP-F2	CTCAAGATCAGAAGTTTCACGCCTGACCTAGACTTAC
	TCH4-GFP-R2	GGTACCCCTAGGCCCTGCAGCTAAGCACTCTTTAGGA
	35S:TCH4-F	GGCGCGCCCACAAACCAATCTAACTCAATACAAG
	35S:TCH4-R	CGGGATCCAACAAAAAAGCACATTGTAACAAAG
	CR-TCH4-F	ATTGCCTTTCACTGCTTCTTACCG
	CR-TCH4-R	AAACCGGTAAGAAGCAGTGAAAGG
qRT-PCR	TCH4-qPCR-F	GCCTCGAAACAGGGGACTAC
	TCH4-qPCR-R	CTTGAGGGAACCTCTTCGCA
	UBQ5-qPCR-F	GTGGTGCTAAGAAGAGGAAGA
	UBQ5-qPCR-R	TCAAGCTTCAACTCCTTCTTT
	eEF1α-qPCR-F	CCTTGGTGTCAAGCAGATGA
	eEF1α-qPCR-R	TGAAGACACCTCCTTGATGATTT
